# Real-life dental examination elicits physiological responses different to visual and auditory dental-related stimuli

**DOI:** 10.1371/journal.pone.0252128

**Published:** 2021-06-03

**Authors:** Tadea Košir, Jakob Sajovic, Maja Grošelj, Aleš Fidler, Gorazd Drevenšek, Polona Selič-Zupančič

**Affiliations:** 1 Faculty of Mathematics, Natural Sciences and Information Technologies, University of Primorska, Koper, Slovenia; 2 Department of Endodontics, University Medical Centre Ljubljana, Ljubljana, Slovenia; 3 Department of Dental Diseases and Normal Dental Morphology, Faculty of Medicine, University of Ljubljana, Ljubljana, Slovenia; 4 Faculty of Medicine, Institute of Pharmacology and Experimental Toxicology, University of Ljubljana, Ljubljana, Slovenia; 5 Department of Family Medicine, Faculty of Medicine, University of Ljubljana, Ljubljana, Slovenia; University of Lausanne, SWITZERLAND

## Abstract

**Background:**

Previous studies on dental anxiety have examined the psychophysiological responses evoked in dentally anxious subjects by dental-related stimuli, but not during a real-life dental examination, which was achieved in the present study.

**Methods:**

The heart rate, skin conductance level, and heart rate variability of 25 subjects with dental anxiety and 25 healthy controls were examined. Anxiety was determined by the Modified Dental Anxiety Scale and the Dental Anxiety Scale–Revised. The psychophysiological reactions of the two groups were compared during exposure to dental-related pictures, dental-related sounds, and an actual examination in a dental surgery.

**Results:**

All the dental-related stimuli provoked an increase in heart rate, i.e. visual stimuli (p<0.001; 95% CI 0.98–3.95 bpm), auditory stimuli (p<0.001; 95% CI 1.34–4.99 bpm), and a dental examination (p<0.001; 95% CI 1.26–5.39 bpm). Dental-related pictures provoked inferior skin conductance level changes compared to dental-related sounds and the dental examination (visual modality *vs* auditory p<0.001; 95% CI 0.039–0.152; visual modality *vs* examination p<0.001; 95% CI 0.083–0.275). Heart rate variability manifested in a complex pattern of responses to the dental examination. However, when exposed to all three dental-related stimuli presentation conditions, the heart rate (F = 0.352, p = 0.556), skin conductance level (F = 0.009, p = 0.926), and heart rate variability parameters of subjects with dental anxiety did not differ in comparison to the healthy controls.

**Conclusions:**

This pilot study represents an evaluation of psychophysiological reactions during a real-life dental examination compared to single modality stimuli, and shows that a real-life dental examination provokes an increase in heart rate, heart rate variability and skin conductance level. Additionally, autonomic responses did not differ between the experimental and control groups. The key issue for future studies is the effect of real-life situations on the physiological and psychological state of the subjects, which should be considered when planning new research and studied in depth.

## Introduction

Dental treatment usually provokes diverse feelings of discomfort, with 10–20% of individuals in the general population experiencing untenable feelings of fear and/or anxiety upon visiting their dentist [1–3].

A disproportionately stressful response to dental procedures is defined as dental anxiety [4]. Unpleasant as it is alone, severe dental anxiety could indicate the deeper issue of dental phobia, with the difference that dental phobia disrupts the daily functioning of the person afflicted, while this is not typical of dental anxiety [5].

In conjunction with the psychological symptoms of fear, discomfort, tension and more, dental anxiety also elicits strong physiological responses [2]. This is due to dental-related stimuli being experienced as threatening in the eyes of the dentally anxious person, causing the activation of evolutionary defence mechanisms, and can be viewed through the lens of the defence cascade model, which describes the evolutionary adaptations of organisms to threats in their environments. A threatened organism mobilizes its survival resources through several changing states, the arousal, flight or fight, freezing, tonic immobility, collapsed immobility, and quiescent immobility [6].

All responses follow an initial state of arousal and sympathetic nervous system activation, comprised of an increase in heart rate (HR), respiration and muscle tone, which is followed by the fight-or-flight response in humans [6–8]. The fight-or-flight response is characterized by a yet further increase in HR, increased perfusion of the organs, and the release of catecholamines [7], which causes a general sympathetic response [6].

In certain situations, be it because of the shock of the situation, its ambiguity, the context or other factors, the fight-or-flight response may be inhibited, and result in ‘freezing’ [6]. Freezing features the inhibition of the motor responses of fight-or-flight, with the addition of the parasympathetic inhibition of cardiac activity [6–8]. Such reactions are usually short, measured in seconds, and evolve into fight-or-flight responses or a gradual return to arousal and then calm, following the passing of the threat [6, 7].

Tonic immobility, collapsed immobility and quiescent immobility all feature strong parasympathetic activation as a response to a severely stressful stimulus, causing the immobility [6]. The variety of different possible reactions to anxious stimuli thus produce variations in both of the branches of the autonomic nervous system [9]. In individuals with dental anxiety, autonomic arousal in a dental-related situation is typically observed as the HR and skin conductance level (SCL) increasing, and the heart rate variability (HRV) decreasing in individuals with dental anxiety [9, 10].

Broadly speaking, two different approaches exploring the autonomic reactivity associated with dental anxiety have been adopted by previous studies [9, 11–16]. The first approach has focused on visual dental-related clues in an effort to discover the reactions typical of dental anxiety and phobia; the results show an increase in the HRV of dentally phobic subjects while observing dental-related stimuli [11], but a decrease in HRV, relative to baseline, for dentally anxious subjects watching video sequences of dental procedures when compared to healthy control subjects (HCs) [9]. In both cases an increased HR was reported, while mixed results were obtained pertaining to the SCL where [11] found no significant differences between the dentally phobic and the HCs, but [9] found an increase in SCL in the dentally anxious while being exposed to visual dental-related stimuli. The second approach investigated autonomic reactivity during the presentation of stimuli in various modalities to better establish which sensory modality provokes greater autonomic reactions to dental-related stimuli.

The comparison of auditory to visual modalities yielded mixed results, the auditory modality was found to be more anxiety-provoking than the visual modality by Hilbert et al. [12]; the authors found increased activation in the phobia-associated regions of the brain, using functional magnetic resonance imaging, in dentally phobic subjects relative to the HCs. Auditory stimuli were corroborated as more anxiety-provoking in a study carried out by Wannemüller et al. [13], where dentally phobic subjects exhibited increased HR during auditory stimuli in comparison to visual ones. Similarly, Oosterink et al. [15] reported that stimuli related to invasive dental procedures were very effective at eliciting anxious responses, with drilling sounds provoking more anxiety than the sight of the drill.

These findings are partially contradicted by Wannemüller et al.’s study [13] where, despite observing a higher HR during auditory stimuli, the dentally phobic subjects rated visual stimuli as more subjectively anxiety-provoking. Extending the discrepancy, a further study showed that individuals with dental phobia assessed visual dental-related stimuli as more anxiety-provoking, while exposure to dental-related pictures provoked increased levels of HR in individuals with dental phobia and decreased levels of HR in the HCs with no differences in HR reactions observed between dentally phobic subjects and the HCs during exposure to dental-related sounds [14].

Other modalities than strictly visual or strictly auditory have rarely been investigated. To our knowledge, only one study has utilized dental-related stimuli in a naturalistic context in the form of a standardized dental examination. Moreover, this examination was carried out in individuals with dental anxiety only, but not in the HCs [16]. This lack of knowledge provokes the question of whether there are any differences in the autonomic response to real-world experiences in comparison to simulated laboratory environments, as there are many factors that differ between real-world and laboratory settings.

These are illustrated by a meta-analysis of neuroimaging studies by Yeung, Goto & Leung [17], who found that auditory and visual stimuli simulating dental treatment do activate the brain regions associated with general anxiety, but fail to activate the amygdala, the main fear processing centre of the brain [18]. This phenomenon is explained by the belief of the participants that dental treatment is not likely to occur in the context of a research environment, and thus does not present a salient enough threat to warrant the arousal of intense fear [17]. A yet further implication of the differences between the settings may be the effect produced by the odours of the chemicals specific to dental treatment procedures discovered by Ninomiya et al. [19], who found activation of the prefrontal cortex, associated with emotional stress and negative emotional reactions, on smelling the resin used in dentistry. An additional factor impacting these differences could be the role of the dentist-patient relationship, which helps patients cope with their anxiety [20–23].

As these studies outline, many factors that are normally absent in laboratory conditions can provoke anxiety in dental patients [16, 17]. A real-life dental examination could potentially elicit different bodily responses in comparison to a simulation study, as it is a complex mix of conditional sensory stimuli, specific to the environment of a dental surgery [16, 18, 19].

The aim of the present study was to provide some preliminary insight into the psychophysiological reactions of subjects with dental anxiety compared to non-anxious subjects when exposed to unimodal dental stimuli and a real-life experience of a dental check-up. The work was carried out as a pilot study and provided a theoretical and practical basis for further research to build upon and further explore the differences between the psychophysiological reactions of people in different dental-related contexts. Reactions in the measured components (HR, SCL and HRV) of the autonomic response were predicted as follows:

H1: The HR of subjects with dental anxiety would increase during exposure to both visual and auditory unimodal stimuli and the dental examination when compared to baseline levels and the HCs. Also in comparison with the HCs, a more pronounced HR reaction of subjects with dental anxiety to auditory stimuli, relative to the visual ones, would be expected, with the dental examination eliciting the largest increase in HR of the three conditions.

H2: A significant increase in SCL would be exhibited by individuals with dental anxiety during exposure to unimodal stimuli and the dental examination, both in comparison with the baseline and the HCs. Just as in HR, the auditory stimuli would be expected to elicit greater changes in SCL than visual ones, and the dental examination would elicit the greatest increase.

H3: HRV would be decreased for subjects with dental anxiety during all three conditions, when compared to baseline levels and the HCs. Again, the auditory stimuli would be expected to elicit a greater response than the visual stimuli with the dental examination eliciting the most pronounced decrease.

## Methods

### Participants

Twenty-five participants with dental anxiety and twenty-five HCs participated in this study. The participants were 18 to 63 years old (M_dental anxiety_ = 44.40 (13.29); M_HC_ = 44.48 (13.07)), and all were Slovenian citizens. The sample was recruited from the community via the snowball method, beginning with dentally anxious acquaintances of the researchers, who in turn recruited more of their own dentally anxious acquaintances into the study. The participants were then screened for dental anxiety; the presence and level of their dental anxiety was evaluated through two different relevant scales, The Modified Dental Anxiety Scale and The Dental Anxiety Scale–Revised. Those meeting the inclusion criteria for dental anxiety were then age matched with the HCs and the sampling was terminated after reaching 25 members in each group. The inclusion criterion for individuals with dental anxiety was set to reach a cut-off score in at least one of the two scales, while the inclusion criterion for the HCs was reaching the cut-off score in neither scale. The exclusion criteria were the presence of cardiovascular diseases or pharmacotherapy affecting the cardiovascular system; having diseases affecting SCL; and psychiatric conditions (except dental anxiety) or current treatment with medication for any psychiatric conditions.

Prior to the execution of the study, the total of 50 subjects was calculated to have more than 90% power to detect a significant association for two-way mixed ANOVA (using an alpha of 0.05, a large effect size of 0.4, two groups and four within-subjects factors) [24].

Given that the research was designed as a pilot study, valuable experience, insights and knowledge was expected to be gained and used in the design of further, more comprehensive research. The study was approved by the Republic of Slovenia National Medical Ethics Committee No. 0120-60/2017.

### Materials

#### Modified Dental Anxiety Scale

The Modified Dental Anxiety Scale (MDAS) is a brief self-report scale used for the assessment of dental anxiety [25]. It consists of five questions related to a dental setting [26]. The participant must respond to each question with one of the given answers, which are scored from 1 to 5. The sum of all five items represents the total score, which ranges from 5 to 25. Anyone who gets a score of 19 or above (the cut-off score) is considered as an individual with high dental anxiety [25, 26]. This scale has been reported to have high reliability and validity [25]. In our study, the MDAS was back-translated from English into Slovene and demonstrated a high level of internal consistency, as determined by a Cronbach’s α of 0.912 in our sample.

#### Dental Anxiety Scale–Revised

The Dental Anxiety Scale–Revised (DAS-R) is composed of four questions concerning the dental setting [27]. Each question offers five answers ranked on a five-point scale. The maximum possible score is 20; scores of 13–14 indicate high anxiety, and scores of 15–20 indicate severe anxiety (or dental phobia) [27, 28]. Its reliability and validity have been reported to be adequate [29]. In our study, the DAS-R was back-translated from English into Slovene and demonstrated a high level of internal consistency, as determined by a Cronbach’s α of 0.956 in our sample.

The consistency between the MDAS and DAS-R scales was tested with Cohen’s kappa. A moderate agreement between the two scales was found: κ = 0.520, 95% CI [0.312, 0.728], p<0.001.

The rationale for using two scales, the MDAS and the DAS-R, for dental anxiety assessment was an effort to yield more reliable results, as both measures are considered to be consistent and clinically useful tools for the assessment of dental anxiety. Additionally, the use of two different scales allowed for the capture of a broader picture of dental anxiety by covering more symptoms in their combined items.

#### Stimuli for visual and auditory modalities

The stimuli were presented to the participants on a personal computer. In the visual modality trial, 10 neutral images were presented in the beginning, each for 6 seconds. After a 10 second break, 10 images related to dental treatment were presented, each for 6 seconds. All the images related to dental treatment were selected by a dentist as representing threatening stimuli, based on their experience in dealing with dentally anxious patients, but were not standardized. The neutral images were selected from the EmoMadrid affective picture database [30] to ensure that the images themselves did not induce elevated levels of arousal.

In the auditory modality trial, the participants were exposed to two types of audio recordings. Firstly, a 60-second recording of neutral sounds (e.g. sounds of traffic and birds) was presented, followed by a 10 second break and then a 60-second continuous recording of sounds related to dental treatment (e.g. sounds of dental procedures). Both audio recordings were custom-made, so the auditory stimuli were not standardized. The presentation of neutral images and sounds was carried out in order for the participants to become accustomed to the task. Psychophysiological reactions were measured during exposure to the visual and auditory dental-related stimuli and during the neutral stimuli of both modalities.

#### Psychophysiological measurement

The Vrije University Ambulatory Monitoring System (VU-AMS, Version 5, Vrije University, Amsterdam) [31] was used to measure autonomic markers during the experimental conditions. The device combines an electrocardiogram (ECG) and measurement of the SCL. The device records the physiological measurements used in this study in the following ranges, with these sample frequencies and impedances:

- ECG: Range of measurement is 11mVp-p, sample frequency is 1 kHz and impedance is >10M Ω.
- SCL: Range of measurement is 0–100μS, sample frequency is 10 Hz and impedance is >2 kΩ.

Skintact F-50 electrodes, Ø50mm with AquaGel and foam backing were used for the ECG recording, and Biopac Skin Resistance Trans TP-TSD203 electrodes in combination with Isotonic 4OZ-GEL101 electrode gel were used for the measurement of SCL. Two Ag-AgCl, non-polarizable Trans TP-TSD203 electrodes, mounted in individual polyurethane housings that terminate in Touchproof connectors, were used. The electrodes were attached to the fingers by Velcro straps and had a 6 mm (diameter) contact area with a 1.6 mm cavity to accommodate the electrode gel. The electrodes were attached according to the Data Analysis and Management Software (DAMS) manual for the VU-AMS. The obtained variables were HR in beats per minute [bpm], SCL in micro Siemens [μS], HRV measured as a standard deviation of all normal RR intervals (SDNN), root mean square of successive differences (RMSSD) in milliseconds [msec], and the ratio of low to high frequency of heartbeat (LF/HF).

The HRV measures were chosen in order to examine the activation of both branches of the autonomic nervous system during the anxiety inducing stimuli presentations and during the dental examination. The SDNN measure provides insight into mixed cyclic sympathetic and parasympathetic effects on the heart, but the effects of the two branches are very hard to distinguish. The RMSSD is the HRV measure most often used in shorter recordings of heart activity, providing the clearest observation of the parasympathetic effect on the HR. The LF/HF measure provides the clearest differentiation of sympathetic and parasympathetic effects on the heart, but is not reliable in the shorter unimodal stimulus presentation conditions [32].

Alongside the HR and SCL measures, the chosen parameters enable us to examine the autonomic nervous system reactions to dental-related stimuli in full, without limitation on a single branch or recording duration.

### Procedure

The study was carried out at the Dental Clinic, University Medical Centre Ljubljana, Slovenia. The same procedure was followed for all the participants: first, the nature of the study was explained and they signed an Informed Consent Form. The procedure consisted of two main parts–psychological and dental. The psychological part was carried out by a psychologist in a room which did not contain any dental-related stimuli. The MDAS and DAS-R scales were administered first, and then the electrodes of the VU-AMS were placed and the participant’s psychophysiological reactions were recorded from that point on. A baseline recording was made for 5 minutes with the subject in an armchair that had a similar incline to a dental chair. The participants were asked to close their eyes and relax. The data from the end of the second minute to the beginning of the fifth were used as a baseline, to allow for accommodation to the supine position at the beginning, and because the final parts of the recording had various movement artefacts. After the baseline measurements, the participants were exposed to the visual stimuli. They were instructed to sit on a chair and observe the sequence of images while being aware of the feelings triggered. Ear plugs were used to ensure that the participants were preferentially exposed to visual stimuli. Afterwards the participants were exposed to the auditory stimuli. The participants were asked to put on headphones and instructed to sit on the chair, close their eyes, listen to the sequence of audio recordings and be aware of the feelings triggered.

In the dental part of the study, the participants were asked to complete a standard medical history questionnaire. The participants were then asked to move to the dental surgery, where a complete dental examination was carried out by a dentist. A thorough medical and dental history was taken, followed by a brief oral examination. The participants were then moved from the sitting to the lying position and a complete intraoral dental examination was made using a dental mirror, sharp dental explorer, periodontal probe, and air/water syringe. First, dental occlusion was determined and the dental soft tissues were examined. Next, dental status, dental caries, and dental plaque were assessed, and the periodontal tissues were examined for signs of gingivitis or marginal periodontitis, such as bleeding on probing and periodontal pockets. On selected teeth, tooth mobility, percussion, and palpation were checked, and a pulp vitality test was carried out. Pulp vitality was confirmed by a thermal pulp vitality test with cold spray (Plurasol Kältespray, Pluradent AG et Co KG, Offenbach, Germany) on a cotton pellet, followed by an electric pulp test with an electric pulp vitality tester (Parkell Pulp Vitality Tester, Farmingdale, USA). As a demonstration, one selected tooth was isolated by a rubber dam. Additionally, dental plaque and calculus were removed using an ultrasonic scaler. Finally, the participant walked back to the room where the experiment began, where the electrodes of the VU-AMS were removed.

### Data and statistical analysis

The DAMS for the VU-AMS was used to process the raw data obtained by the VU-AMS device. All the ECG and SCL signals were averaged separately for each of the four experimental conditions, and automatically scored and calculated into HR, SCL, SDNN, RMSSD and LF to HF ratio variables by the DAMS program. The LF to HF ratio was calculated only for the baseline and dental examination conditions, due to the limitation of the other conditions containing less than 4 minutes of recording, making this measure unreliable [31].

In order to further explore the effects of dental anxiety, correlations of all measures with the DAS-R and the MDAS scales were calculated. The results of correlations provide additional insight into the associations between self-reported measures of dental anxiety and physiological reactions to dental-related stimuli.

The data were prepared in line with the DAMS manual for the VU-AMS (VU-AMS, version 5, Vrije University, Amsterdam) [31]. For the ECG data, automated artefact labelling was used to identify clipping and signal loss, while suspicious peaks and other possible artefacts were visually inspected and manually corrected if required. The SCL signal was pre-processed to remove noise and power line interference in the signal. A low pass filter with a cut-off frequency of 2 Hz was used to filter the signal. In order to avoid shifting of peaks, filtering was done in both the forward and reverse directions.

The Statistical Package for the Social Sciences (SPSS, 22; IBM Corporation, New York) was used to conduct all statistical analyses, a p<0.05 significance level was adopted. A Mann-Whitney U test was conducted to determine differences in age and the MDAS and DAS-R scores between the dental anxiety group and the HCs. Four separate 2-way mixed analyses of variance (ANOVA) were run, one for each dependent variable meeting the assumptions, with the independent variables experimental group as a between-subject factor, and experimental condition as a within-subjects factor. No outliers were detected. Shapiro-Wilk’s test showed non-normal distributions for the MDAS, DAS-R, SCL and SDNN data. Levene’s test demonstrated inhomogeneous variance of the SDNN visual modality and dental examination data, as well as baseline LF/HF data. Box’s test indicated inhomogeneous covariance for the RMSSD and LF/HF data. A logarithmic transformation with a natural logarithm was used for the SCL and LF/HF data and a square root transformation for the SDNN data. Both the SDNN and the SCL data were centred [33] before performing their respective transformations. The assumption of sphericity was violated in the case of the HR, SCL and RMSSD data. The Greenhouse Geisser correction was therefore applied when required. The pairwise comparisons were all Bonfferoni-adjusted and were reported with 95% confidence intervals.

As the LF/HF and RMSSD data did not meet the assumptions of the ANOVA, non-parametric tests were run to analyse these variables, although in the case of the LF/HF data a repeated measures ANOVA was also performed, as the assumption of equality of covariances was barely violated. The RMSSD data was thus evaluated using the Friedman test with post-hoc Wilcoxon Signed-Rank tests corrected with the Bonfferoni adjustment for multiple comparisons, while the LF/HF data were additionally evaluated by the Wilcoxon Singed-Rank test. Non-parametric correlations using the Kendall’s Tau-b correlation were also calculated between all parameters and the DAS-R and MDAS scales.

## Results

### Sample characteristics

Table 1 summarizes the demographic and clinical characteristics of the sample. The dentally anxious group achieved significantly higher scores than the HCs on both the MDAS and DAS-R questionnaires (U = 611.5, z = 5.824, p<0.0005 and U = 625.0, z = 6.087, p<0.0005, respectively). According to the MDAS, 12 participants in the dentally anxious group were classified as having high levels of dental anxiety or dental phobia. According to the DAS-R seven participants in the dentally anxious group showed high levels of dental anxiety and 18 showed severe dental anxiety or dental phobia. The dentally anxious group and HC group did not differ in age. Of all the participants, 36 were women and 14 men. The analyses for the LF/HF and RMSSD parameters were performed on 49 subjects, as one data set was lost due to data corruption in the course of the analyses.

**Table 1:** Demographic and clinical characteristics of the sample.

	DA group (n = 25) M±SD	HC group (n = 25) M±SD
**Age**	44.40 ±3.29	44.48±13.07
**Age (Median and IQR)**	51 (18)	47 (19)
**MDAS**	18.44±3.22	8.80±3.20
**DAS-R**	16.00±2.18	7.68±2.25

*DA*: *Dental Anxiety; HC*: *Healthy Control; MDAS*: *Modified Dental Anxiety Scale; DAS-R*: *Dental Anxiety Scale–Revised*, *IQR*: *Inter-Quartile Range*

### Heart rate

**Fig 1:**
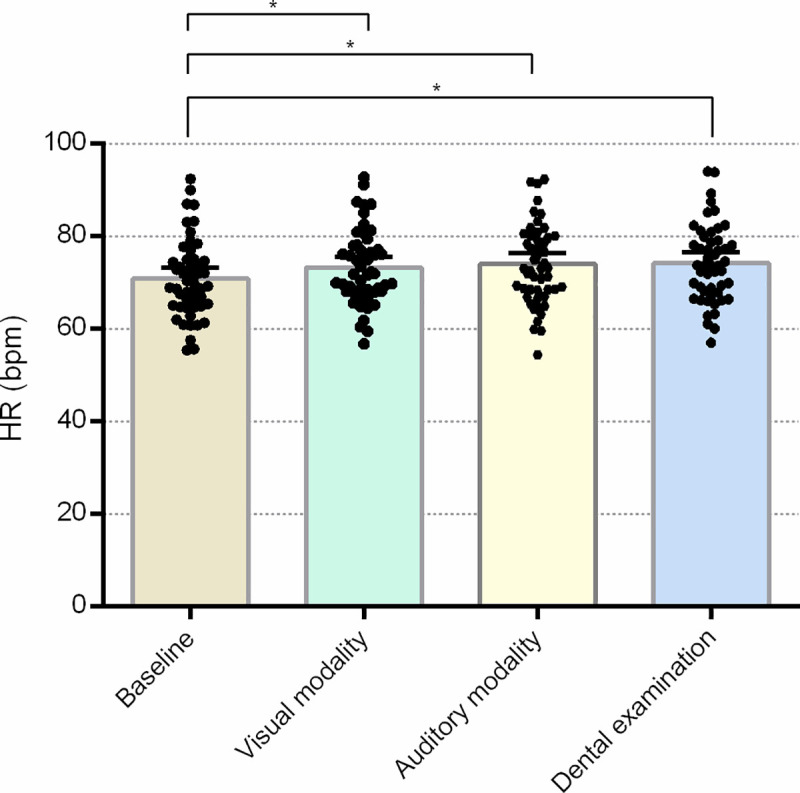
Mean heart rate during different experimental conditions. Error bars indicate a 95% confidence interval; * p<0.001; units are in bpm (beats per minute).

The main effect of the experimental condition was statistically significant (F = 11.422; p<0.001; partial η^2^ = 0.192); a significant increase in HR of 2.47 bpm was identified (95% CI 0.98–3.95; p<0.001) compared to the baseline HR parameter (M = 70.86±8.51 bpm) during exposure to visual stimuli (M = 73.33±8.09 bpm), auditory stimuli (increase of 3.17 bpm (95% CI 1.34–4.99; p<0.001)), and during the dental examination (significant increase of 3.27 bpm (95% CI 1.26–5.39; p<0.001)). Neither the group effect (F = 0.352, p = 0.556, partial η^2^ = 0.007), nor the interaction (F = 1.645, p = 0.193, partial η^2^ = 0.033) were statistically significant. Non-significant results of HR analyses are presented in detail in the S1 Appendix. An overview of the HR results is provided in Fig 1.

### Skin conductance level

**Fig 2:**
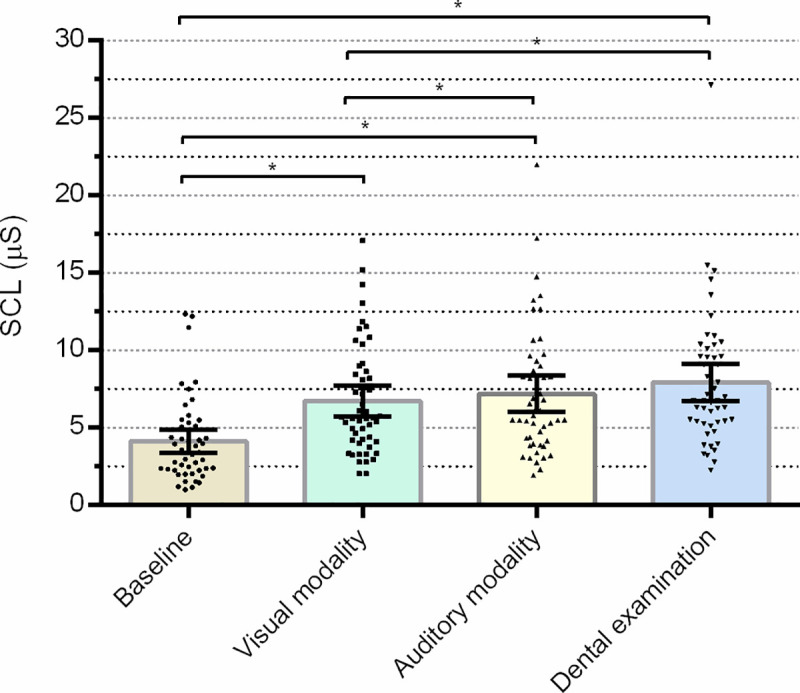
Mean skin conductance level during different experimental conditions. Error bars indicate a 95% confidence interval; * p<0.001; units are in μS (micro Siemens).

A significant main effect of the experimental condition in the mean SCL parameter was identified (F = 55.900; p<0.001; partial η^2^ = 0.538). A pairwise comparison showed an increase in the SCL parameter during exposure to visual stimuli compared to baseline levels (p<0.001; 95% CI -0.430-(-0.172)), during exposure to auditory stimuli (p<0.001; 95% CI -0.525-(-0.267)), and also during the dental examination (p<0.001; 95% CI -0.608-(-0.353)). There was an increase in SCL during exposure to auditory stimuli compared to visual stimuli (p<0.001; 95% CI 0.039–0.152), and an increase in the SCL parameter during the dental examination compared to visual stimuli (p<0.001; 95% CI 0.083–0.275). Fig 2 represents the significant results of the pairwise comparison of non-transformed data. The effects of group (F = 0.009, p = 0.926, partial η^2^ = 0.00018) and interaction (F = 2.117, p = 0.101, partial η^2^ = 0.042) proved to be non-significant. Other non-significant results of SCL analyses are presented in S2 Appendix.

### Standard deviation of all normal R-R intervals

**Fig 3:**
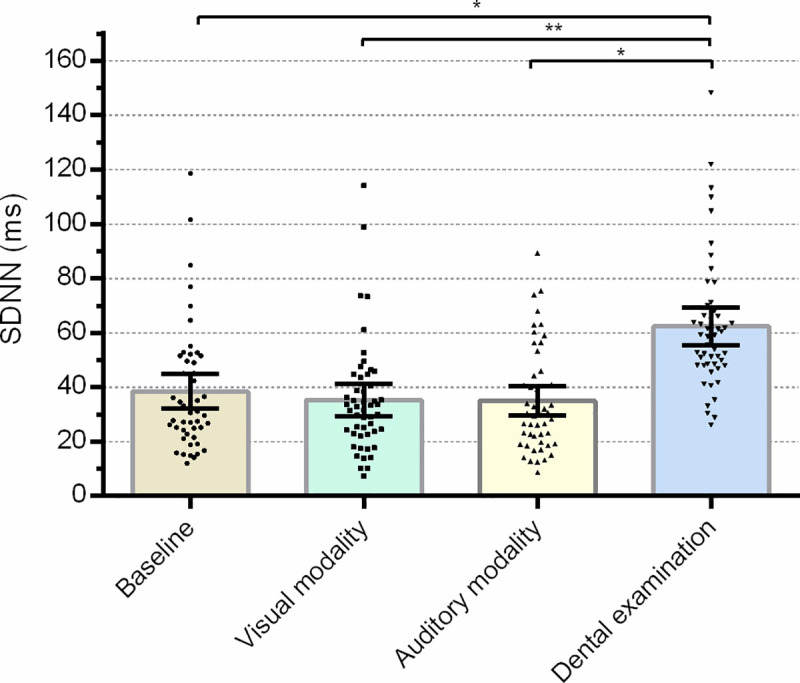
Mean heart rate variability as SDNN during different experimental conditions. Error bars indicate a 95% confidence interval; * p<0.001; ** p<0.05; units are in milliseconds.

The main effect of the experimental condition was statistically significant (F = 9.345; p<0.001; partial η^2^ = 0.163), as was an increase in the SDNN parameter during the dental examination compared to baseline (p = 0.001; 95% CI 0.345–1.574), and in the SDNN parameter during the dental examination compared to the visual modality SDNN (p = 0.005; 95% CI 0.168–1.266). An increase in SDNN during the dental examination in comparison to the auditory modality SDNN was also identified (p = 0.001; 95% CI 0.288–1.462). Fig 3 shows the significant results of the pairwise comparison of non-transformed data. Neither group (F = 0.129, p = 0.721 partial η^2^ = 0.003) nor interaction (F = 2.402, p = 0.070, partial η^2^ = 0.048) effects were significant. Non-significant results of the SDNN analyses are further presented in the S3 Appendix.

### Root mean square of the successive differences

**Fig 4:**
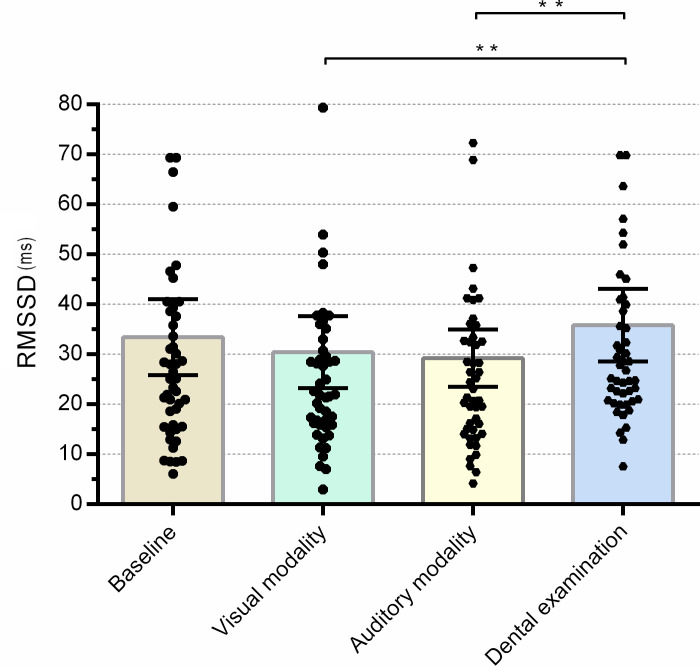
Mean heart rate variability as RMSSD during different experimental conditions. Error bars indicate a 95% confidence interval; ** p<0.05; units are in milliseconds.

The effect of the experimental condition was statistically significant (χ^2^ = 15.294; p = 0.002). The differences between the RMSSD during the auditory modality compared to the dental examination (Z = -2.611; p = 0.054), and during the visual modality compared to the dental examination (Z = -2.601; p = 0.054), were on the edge of statistical significance; the RMSSD were lower during the auditory and visual modality than during the dental examination. Group effects proved to be non-significant (F = 0.729, p = 0.399, partial η^2^ = 0.015). Other non-significant results of the RMSSD analyses are presented in the S4 Appendix. In Fig 4 an overview of the RMSSD results, organized by experimental conditions, is presented.

### Low to high frequency ratio

**Fig 5:**
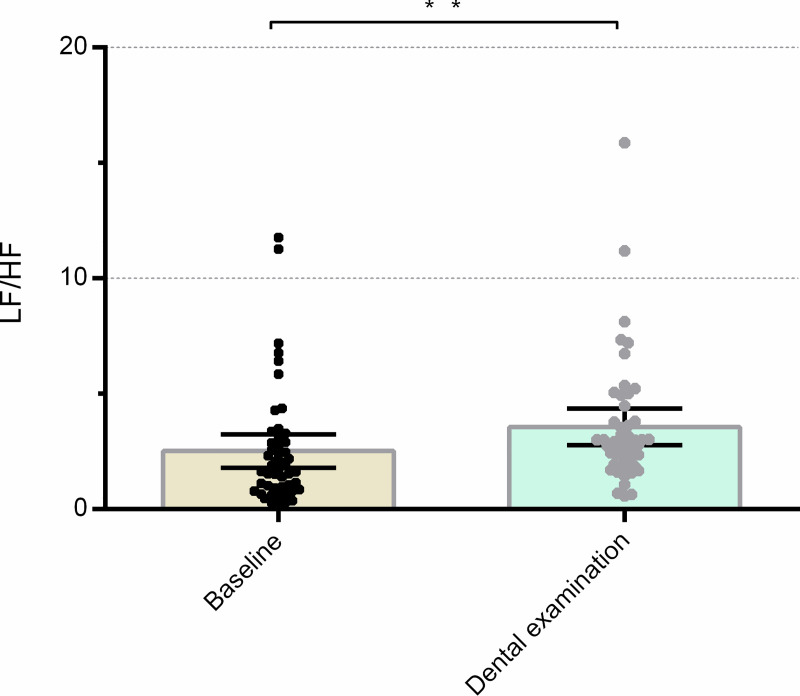
Mean heart rate variability as LF/HF during the baseline and the dental examination. Error bars indicate a 95% confidence interval; ** p<0.05.

The main effect of the experimental condition was statistically significant (F = 18.705; p<0.001; partial η2 = 0.285) and corroborated by the Wilcoxon signed ranks test (Z = -3.626; p<0.001). There was an increase in the LF/HF ratio during the dental examination in comparison to the baseline (p<0.001; 95% CI 0.287–0.786). The group (F = 0.076, p = 0.784, partial η^2^ = 0.002) and interaction (F = 0.025, p = 0.875, partial η^2^ = 0.001) effects were non-significant. Detailed non-significant results of the LF/HF analyses are presented in the S5 Appendix. For a representation of LF/HF results in the baseline and dental examination condition see Fig 5 below.

**Fig 6:**
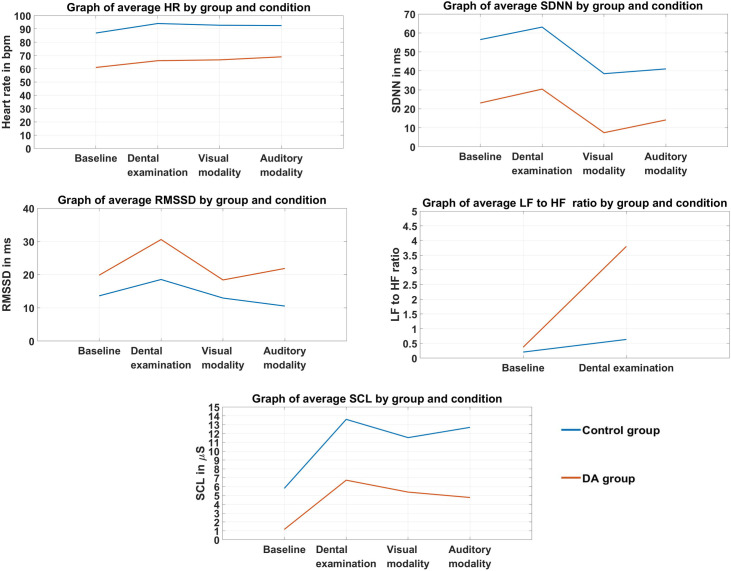
A summary of the results. All of the measured parameters are presented in Fig 6 for ease of visual comparison of group responses to different stimulus modalities and the real-life dental examination. DA group = dentally anxious group. Note that the plotted data is non-transformed.

Fig 6 provides a graphical summary of the results, separately for the HCs and subjects with dental anxiety and also organized by experimental condition. Statistically significant differences are not marked in Fig 6, for those please see Figs 1–5.

### Correlations of measures and scales

The results of correlations are presented in detail in the S6 Appendix. In summary, the two scales, the MDAS and DAS-R were significantly positively inter-correlated (Kendall’s tau-b = 0.736; p<0.001). When examining the correlation of the two scales with the HR parameter, only the HR during the auditory modality of stimulus presentation was correlated with the DAS-R score. No other significant correlations with HR were observed, neither for the DAS-R nor MDAS. Correlating the two scales with the SCL in all conditions, the two scales were not significantly correlated with the SCL, neither were they significantly correlated with the SDNN, RMSSD nor the LF/HF in any condition (S6 Appendix).

Aside from this, the HR during the baseline, the visual modality, auditory modality and the dental examination were also significantly inter-correlated (p<0.001) (S6 Appendix). The same is true of the SCL, SDNN and the RMSSD, which were significantly inter-correlated in all conditions (all p<0.001) (S6 Appendix). The LF/HF during the baseline measurement was significantly positively correlated with the LF/HF during the dental examination (Kendall’s tau-b = 0.262; p = 0.008) (S6 Appendix).

## Discussion

This study investigated the psychophysiological reactions measured by the HR, SCL, SDNN, RMSSD and LF/HF parameters in individuals with dental anxiety and HCs during exposure to visual, auditory, and real-life dental-related stimuli. The main findings are:

1. Exposure to the dental examination, relative to baseline measurements, provoked changes in the psychophysiological reactions of all participants (Figs 1–3, 5 and 6), except for the RMSSD parameter (Fig 4, S4 Appendix).
2. The psychophysiological reactions of subjects with dental anxiety and the HCs did not differ significantly during exposure to the two one-modality stimuli and the dental examination (S1–S5 Appendices).

Examining the effects of stimuli presentation conditions alone, the parameters of HR, SCL, SDNN and LF/HF significantly differed (Figs 1–3 and 5). This shows that the selection of the dental-related stimuli was appropriate, as they were able to elicit physiological responses in the study subjects. A significant drop in the HR parameter obtained during baseline measurements relative to exposure to all three types of stimuli, and an elevated SCL reaction to the auditory stimuli and examination in comparison to visually presented stimuli had already been found in previous studies [9, 12, 15]. The expected increase in average LF/HF observed during the dental examination (Fig 5, S5 Appendix) [34] reflects a greater sympathetic activation of the nervous system. The observed increase in the HR and SCL parameters in all three stimulus presentation contexts is congruent with the activation of the arousal phase of the defence cascade [6, 7]. Moreover, the dental examination was shown to be the experimental condition eliciting the largest changes in psychophysiological parameters that increased during the dental examination when compared to baseline measurements. When the real-life dental examination was compared to the visual and auditory stimulus conditions, a greater SCL and SDNN was observed than during the visual stimuli exposure, and a greater SDNN during the examination than during the auditory stimulus presentation. These results for the SDNN measure could reflect an increase of activation of both branches of the autonomic nervous system during the dental examination [32].

However, the results showed no major differences in HR during the three conditions of dental-related stimuli exposure (S1-S3 Appendices) [13–15]. This and equal sweating response to the dental examination and the auditory stimulus modality (S2 Appendix) [15] as well as the SDNN increasing the most during the dental examination were in disagreement with previous research [15]. The findings from the analysis of the RMSSD data were also unexpected [35], as the only differences identified were between the mean RMSSD during the auditory and visual modalities in comparison with the dental examination (Fig 4, S4 and S6 Appendices). The increase of the RMSSD during the dental examination could represent more interplay between the two branches of the autonomic nervous system, increasing variability of heart rate due to their mixed effects. In comparison, less ambiguous unimodal stimuli thus resulted in a lower RMSSD, as less interference of varied context dependent stimuli allowed for better parasympathetic control of the heart [32].

Group comparisons show that the two groups, subjects with dental anxiety and HCs, did not differ in any of the measures and conditions, which is an unexpected result of this study. HR results contradict the findings of previous studies, where differences in the HR responses of individuals with dental phobia and the HCs were detected [13, 14]. The same holds true for the SCL and HRV parameters, which were found to be different in dentally phobic participants when compared to the HCs in [11] (S3 and S4 Appendices).

The unexpected findings of condition effects may be explained by the complexity of human anxious responses, as the dental examination successfully triggered significant changes from baseline in the SDNN, HR and SCL parameters (S1-S3 Appendices), but not the RMSSD (S4 Appendix), the measure with the least mixed effects of parasympathetic and sympathetic systems. With multiple simultaneous stimulus modalities at play and their interactions with cognitive, environmental and psychological factors, this complexity of autonomous and conscious reactions was shown to be relevant in semi-naturalistic environments. It was also demonstrated by no characteristic fight-or-flight behaviours being identified, presumably due to the successful inhibition of the fight-or-flight response in this study’s subjects, regardless of their dental anxiety [6]. The context of stimulus presentation and the participants’ awareness that no matter what happened it would not feature very invasive procedures could have variably affected the different parameters [17].

Eliciting changes in more parameters than the one-modality visual and auditory stimuli, the most complex anxious response could be attributed to the dental examination, with it being evaluated as more salient a threat than unimodal stimuli, which is similar to the findings of Yeung et al. [17] with regard to the failure of one-modality visual and auditory stimuli to produce the levels of amygdala activation typical of the anxious condition.

Unexpected HRV results could further be explained by a cycle of arousal and freeze responses. This cycle began with initial arousal due to dental-related stimuli being presented. Next, after inhibition of the fight-or-flight response, the subjects entered the freeze response [7, 8], common during states of anticipatory anxiety elicited by the presented stimuli and the dental examination [6–8]. Through the process of habituation to stimuli, the subjects gradually came out of the frozen state and returned to a state of increased vigilance, as the dental stimuli presented were assessed as not life-threatening [6, 7]. The state of freezing could be elicited anew by, for example, a change in the instruments used, such as the transition from a dental probe to the use of a pulp vitality tester, which changes the modality of the stimuli from auditory and tactile to the odours produced by the materials used, in addition to the different tactile and auditory stimuli produced by the change in equipment. HRV would thus increase as the subjects transitioned from a state of arousal, with HR heightened by the sympathetic nervous system mobilizing energy reserves for a response to the threat, to the freezing state, with the parasympathetic nervous system slowing the HR in response to more threatening stimuli [6–8]. The lower RMSSD during the auditory and visual modalities (Fig 4, S4 and S6 Appendices) could also reflect this cycle as being less prominent during the less salient unimodal stimuli compared to the examination. Other findings (S4 Appendix) of this study make it unlikely the RMSSD result was due to the unimodal stimuli being more threatening. The change from lower to higher average LF/HF ratio during the dental examination (Fig 5, S5 Appendix) further supports the cycle hypothesis, as it marks greater sympathetic activity [34].

The inconsistency with previous studies regarding the absence of difference between the control and experimental group may be threefold:

1. This discrepancy could have occurred due to individuals with dental anxiety being included, while in other studies the participants were mostly individuals with dental phobia.
2. These findings may be attributed to the expertise of the dentist, who established an adjusted dentist-patient relationship, which is an important aspect of anxiety management [20–23].
3. A common feature of participants, as trait anxiety affects the HRV and the high frequency power of HR when comparing individuals with high and low trait anxiety [36], given that anxious traits are known to modulate psychophysiological reactions. Weaker, less salient stimuli and smaller differences in trait anxiety give rise to smaller differences in psychophysiological reactions [36–38].

Although multiple efforts were made to eliminate possible methodological errors, some limitations still occurred:

1. The use of a blocked design in the presentation of neutral and dental-related stimuli, which provoked the overall activation of the autonomic nervous system during the experiment, instead of discrete reactions to each of the presented stimuli. Instead, random presentations of dental-related stimuli would yield an experiment that would be more robust to habituation and expectation effects. We should consider that the participants were aware of being at the dental clinic and were exposed to sensory stimuli typical of the clinical environment. Therefore it would have been beneficial for our baseline measurements and measurements during exposure to visual and auditory stimuli to be conducted outside the dental clinic and not on the same day as the dental examination was carried out.
2. It would have been prudent to classify an individual as having dental anxiety when they achieved a cut-off score not only on the DAS-R but also on the MDAS scale.
3. Specific to the HRV parameters used in this study, visual and auditory stimuli presentations should be extended, to allow for the reliability of the LF/HF parameter. The SDNN parameter could have been affected by environmental sounds and thus raised [39]. The possibility of elevated SDNN due to adaptation to stressful conditions should also not have been ignored [35, 40–42], although an increase above baseline is unlikely.
4. On the topic of data quality, the violation of the assumption of homogeneity of variances in some parameters might be a drawback. The equality of covariance assumption was violated for the LF/HF data, but the findings from the ANOVA were corroborated by the Wilcoxon signed ranks test, ameliorating some of the drawbacks (S5 Appendix).
5. Although no time was allotted to accommodation to the supine position at the start of the dental examination, it should not be considered as a major omission as arrhythmias during tilt-table tests usually last between 10 and 15 seconds [43–45].

This study contributes to the body of research, being the first attempt at examining psychophysiological reactions during an actual dental examination and deepens the understanding of dental anxiety. To achieve even better understanding of dental anxiety, future research should focus on an integrative approach, exploring psychophysiological reactions and neural correlates combined with a personal evaluation of specific sections of the dental examination, in order to provide information about which sections of the dental examination should be adjusted in order to make a dental visit less threatening.

## Supporting information

S1 Appendix Results of statistical analyses of HR data. (TIF)

S2 Appendix Results of statistical analyses of SCL data. (TIF)

S3 Appendix Results of statistical analyses of SDNN data. (TIF)

S4 Appendix Results of statistical analyses of RMSSD data. (TIF)

S5 Appendix Results of statistical analyses of LF/HF data. (TIF)

S6 Appendix Results of questionnaire correlations and result analyses. This appendix contains the correlation tables for the questionnaires and psychophysiological variables and the comparison of the two groups by their results on both questionnaires by a Mann-Whitney U test.
(TIF)
