# Supplementary figures and images for: Real-life dental examination elicits physiological responses different to visual and auditory dental-related stimuli
Source: PLoS One. 2021 Jun 3;16(6):e0252128. doi: 10.1371/journal.pone.0252128 (PMC8174713; doi:10.1371/journal.pone.0252128)

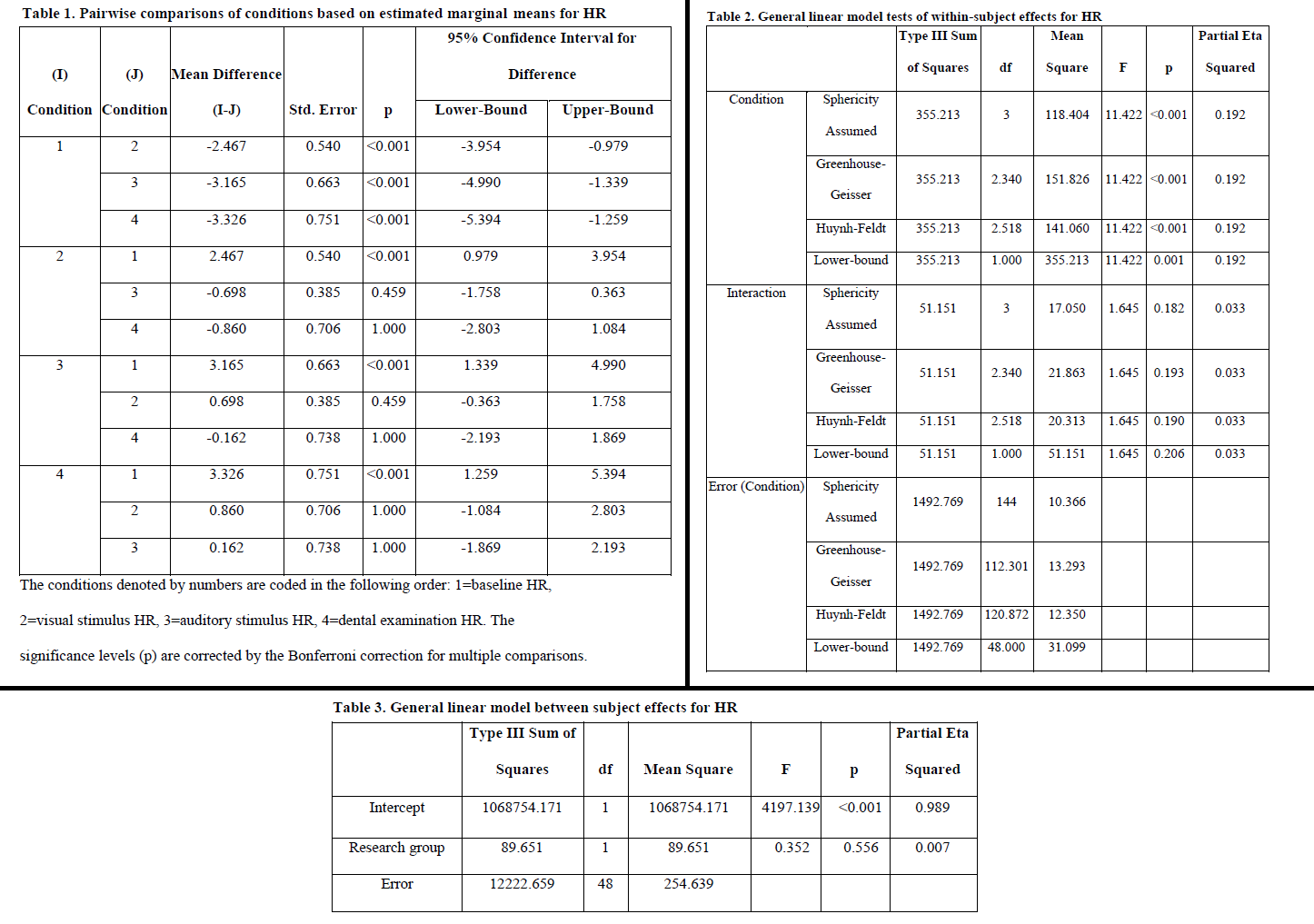

Supplement: S1 Appendix — (TIF) [file pone.0252128.s001.tif]

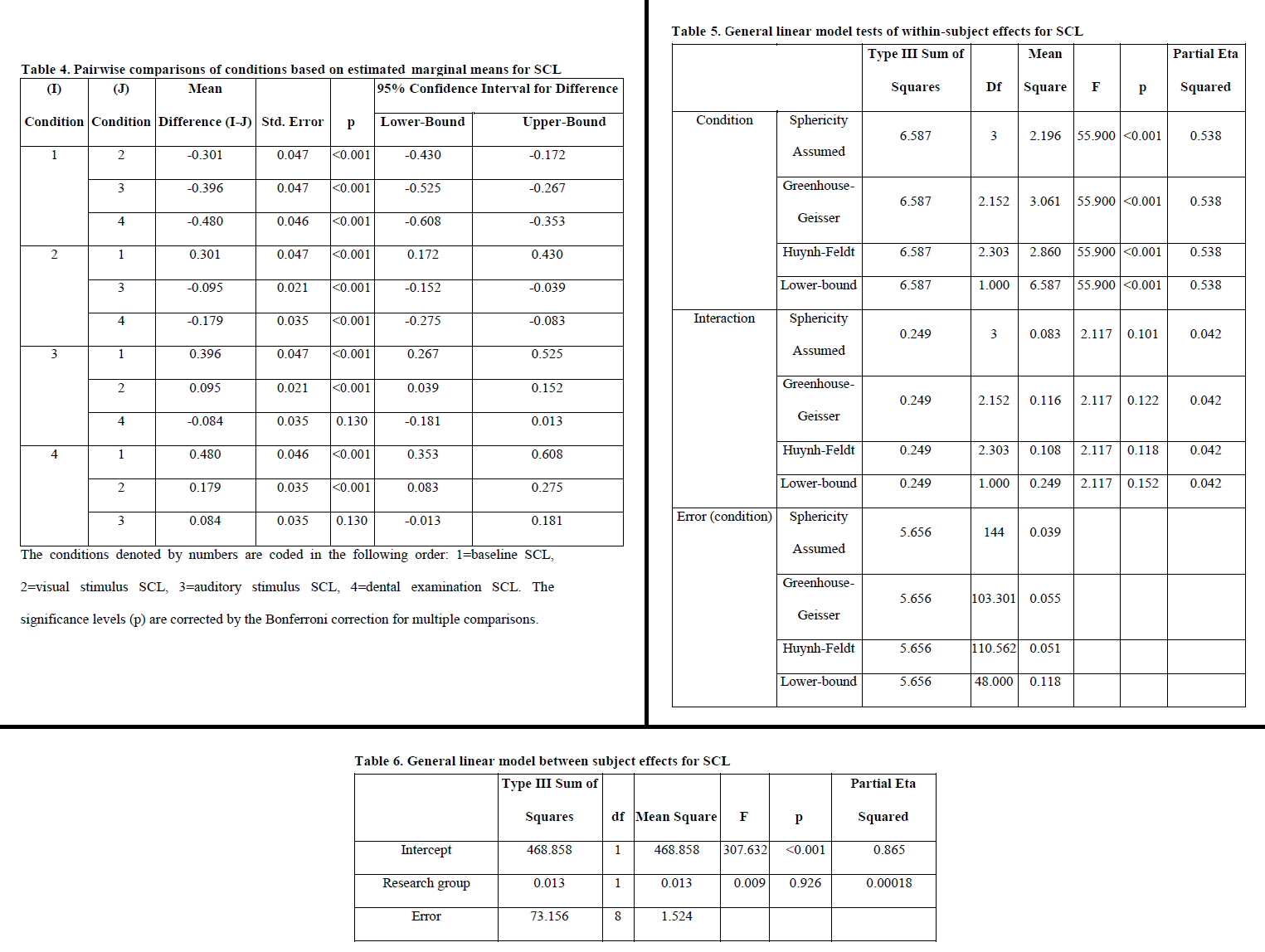

Supplement: S2 Appendix — (TIF) [file pone.0252128.s002.tif]

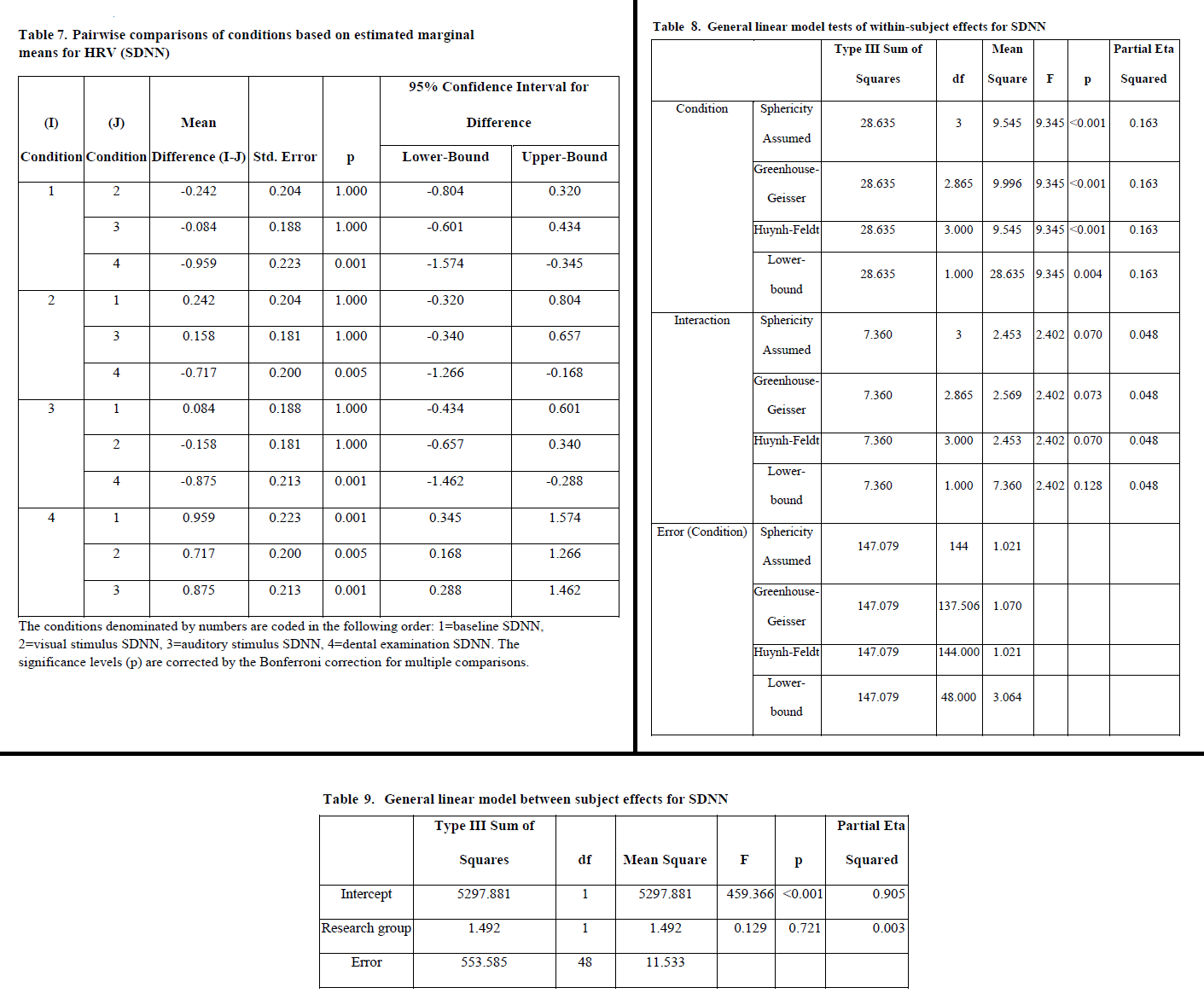

Supplement: S3 Appendix — (TIF) [file pone.0252128.s003.tif]

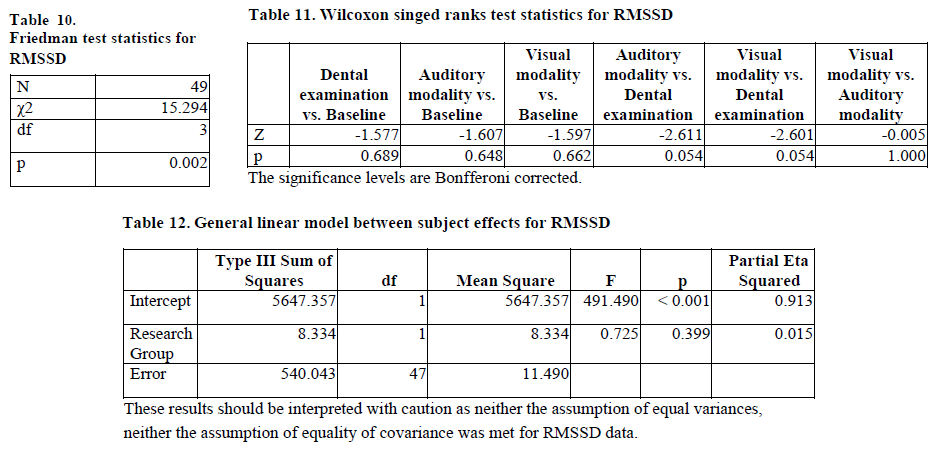

Supplement: S4 Appendix — (TIF) [file pone.0252128.s004.tif]

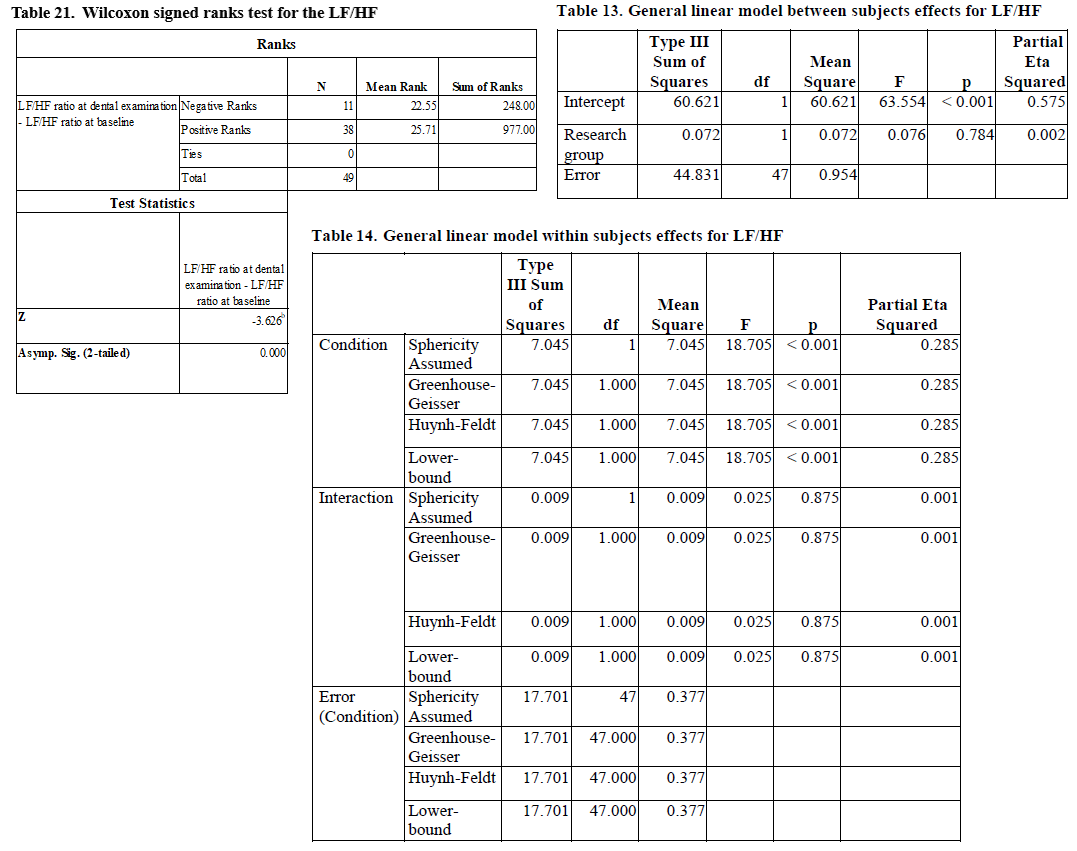

Supplement: S5 Appendix — (TIF) [file pone.0252128.s005.tif]

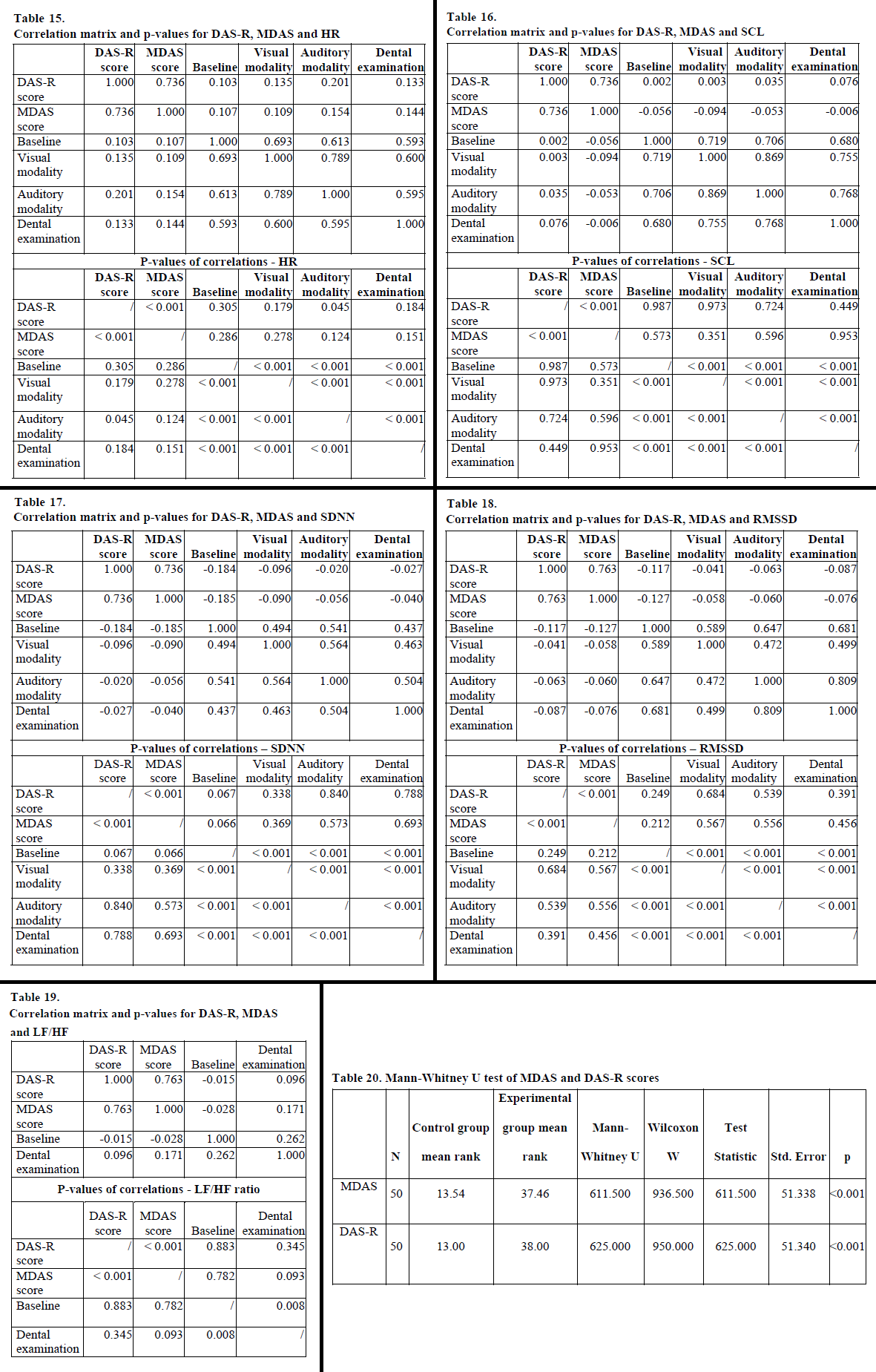

Supplement: S6 Appendix — This appendix contains the correlation tables for the questionnaires and psychophysiological variables and the comparison of the two groups by their results on both questionnaires by a Mann-Whitney U test. (TIF) [file pone.0252128.s006.tif]
